# From medical strategy to foodborne prophylactic strategy: Stabilizing dental collagen with aloin

**DOI:** 10.1002/fsn3.3795

**Published:** 2023-11-07

**Authors:** Chongzhi Jia, Hua Li, Zhongliang Yang, Rongchen Xu, Lijun Wang, Hongbo Li

**Affiliations:** ^1^ Department of Stomatology, The First Medical Center Chinese PLA General Hospital Beijing China; ^2^ Department of Stomatology, The Third Medical Center Chinese PLA General Hospital Beijing China

**Keywords:** aloin, dental collagen, foodborne prophylactic strategy, hydrogen bonds, stability

## Abstract

Infectious oral diseases are longstanding global public health concerns. However, traditional medical approaches to address these diseases are costly, traumatic, and prone to relapse. Here, we propose a foodborne prophylactic strategy using aloin to safeguard dental collagen. The effect of aloin on the stability of dental collagen was evaluated by treating dentin with a solution containing aloin (0.1 mg/mL) for 2 min. This concentration is comparable to the natural aloin content of edible aloe. Furthermore, we investigated the mechanisms underlying the interactions between aloin and dentin collagen. Our findings, obtained through fluorescence spectroscopy, attenuated total reflection Fourier transform infrared spectroscopy, Gaussian peak fitting, circular dichroism spectroscopy, and X‐ray diffraction, revealed that aloin interacts with dental collagen through noncovalent bonding, specifically hydrogen bonding in situ. This interaction leads to a reduction in the distance between molecules and an increase in the proportion of stable α‐helical chains in the dental collagen. The ultimate tensile strength and thermogravimetric analysis demonstrated that dental collagen treated with aloin exhibited improved mechanical strength and thermostability. Additionally, the release of hydroxyproline, cross‐linked carboxy‐terminal telopeptide of type I collagen, and C‐terminal cross‐linked telopeptide of type I collagen, along with weight loss, indicated an enhancement in the enzymatic stability of dental collagen. These findings suggest that aloin administration could be a daily, nondestructive, and cost‐effective strategy for managing infectious oral diseases.

## Graphical abstract

In this study, a foodborne prophylactic strategy with 1.0 mg/mL aloin was developed with the capacity to interact with dental collagen via hydrogen bonds to enhance dental collagen stability. This strategy can increase the proportion of α‐helix of dental collagen. The mechanical strength, thermostability, and the enzymatic stability of dental collagen can be improved with the use of aloin as a daily strategy.

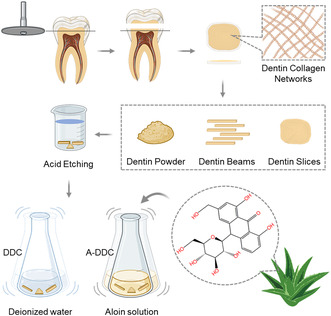

## 1 INTRODUCTION

Infectious oral diseases, including caries, pulpitis, and periapical periodontitis, have been a significant global public health issue for decades (Guo et al., 2019; Liu, Zong, et al., 2022; Omar et al., 2022). In many low‐ and middle‐income countries, a large number of patients are unable to afford the necessary medical expenses for treating these oral diseases (Benzian et al., 2021; Ghahramani et al., 2022). In the presence of bacteria and enzymes, as well as changes in temperature, apatite mineral crystals in the enamel and dentin gradually deteriorate, exposing the underlying organic dental collagen network (Wang, Wang, et al., 2022). When dental collagen is exposed, it becomes more vulnerable to external infections, and its triple‐helix structure deteriorates rapidly (Nowaczyk et al., 2021). Loosening of the collagen structure and the high porosity of the collagen network facilitate the diffusion of infectious substances. As the disease progresses, the infection can spread to the dental pulp and cause bacteria to enter the blood circulation, leading to local infections, such as cellulitis and osteomyelitis of the jaw, or even systemic infections, such as sepsis and multiple organ failure (Dudek‐Wicher et al., 2022; Sabharwal et al., 2021). Therefore, the prevention and treatment of infectious oral diseases are of immense clinical significance with far‐reaching consequences.

Dentists commonly use drills for mechanical grinding to remove infected dental tissue. Subsequently, a dental bonding strategy was proposed to physically separate dental collagen from exogenous infectious agents and to fix dental prostheses made of composite resin, metal, or zirconia. During this process, the adhesive resin can penetrate the dentin collagen network and wrap it with collagen fibrils through micromechanical interlocking, ensuring a strong connection between the restoration and the dental tissues (Breschi et al., 2018). Although current treatment methods are widely acknowledged, it is important to note that they have certain limitations. First, dentists must expand the scope of mechanical grinding to effectively eliminate infectious tissues (Wu et al., 2019). Nevertheless, this approach contradicts the objective of preserving as much natural tooth tissue as possible because of traumatic experiences. Second, bonding interfaces, especially dentin collagen, are vulnerable to moisture, bacteria, and biomechanical factors (Del Carpio‐Perochena et al., 2022; Ho et al., 2018; Yu, Zhao, et al., 2022). Some studies have proposed to rebuild the “inorganic–organic” hybrid structure of dentin through remineralization to enhance the stability of dental collagen (Liu, Hao, et al., 2021). Scientists have used amorphous mineral precursor nanoparticles, such as bioactive glass, calcium phosphate, and silica, to reproduce the mineralization process (Abuna et al., 2021; Shi et al., 2019; Yu, Bian et al., 2022). However, the remineralization period is so long that it cannot be used in clinical and daily applications (Qu et al., 2020). The introduction of antibacterial agents, such as quaternary ammonium salts, metal ions (such as Ag^+^, Cu^2+^, and Zn^2+^), and antibiotics, can strongly resist bacterial infection (Wang, Liu, et al., 2022; Zenga et al., 2022). However, toxicity and drug resistance limit its use. Third, conventional strategies are typically reserved for patients who have already experienced tooth tissue infections or dental defects. For individuals at high risk but currently without any infection, such as those on a high‐carbohydrate diet or patients with temporomandibular disorders, excessive occlusal forces, or tooth wear, this method can lead to more extensive damage and an increased risk of secondary infection (Lam et al., 2020; Murtaza et al., 2019). Furthermore, all the strategies mentioned above must be executed by professional dentists and cannot be applied universally by the general population. Hence, there is an urgent need to develop a low‐cost, nondestructive, effective, and daily strategy to enhance the stability of collagen.

Modifying collagen using exogenous agents is a noninvasive approach that can enhance the structural stability of dentin collagen. Synthetic agents like glutaraldehyde have been proposed to improve collagen stability (Tian et al., 2015). However, their toxicity renders them unsuitable for regular medical applications (Maravic et al., 2018). Water molecules can competitively bind to the active groups (–OH and –NH_2_) of dental collagen, restricting the chemical interaction between these agents and collagen (Jee et al., 2016; Natarajan & Kiran, 2019). Natural agents, including tangerine, procyanidins, and hesperidin, are candidates for enhancing dental collagen structure in humid environments due to the presence of catechol groups in their chemical structures (Islam et al., 2012; Li, Yao, et al., 2021; Riaz et al., 2023). Additionally, they exhibit improved biocompatibility and biosecurity (Nag & Majumder, 2022; Yamakoshi et al., 2002). Aloin, derived from the natural edible aloe, has gained popularity owing to its diverse pharmacological activities, such as antitumor (Zhang et al., 2017), anti‐inflammatory (Jiang et al., 2018), antiosteoporotic (Zhang et al., 2020), and antiviral properties (Lewis et al., 2022). Aloin shares similarities with plant‐derived polyphenols, such as procyanidins, as it contains catechol groups. Thus, aloin has the potential to be a candidate for a foodborne, noniatrogenic, preventive, and therapeutic strategy for patients or individuals at high risk because it can be ingested directly (Yang et al., 2022). However, confirmation is required to determine whether natural edible aloins enhance dentin collagen stability. The study aimed to investigate the effects of aloin on the in situ stability of dental collagen. Additionally, we aimed to explore the mechanisms underlying the interaction between aloin and dentin. The null hypothesis was that the application of aloin would not improve the stability of dental collagen.

## 2 MATERIALS & METHODS

### 2.1 Reagents and dental collagen preparation

Aloin (CAS: 8015‐61‐0) and phosphoric acid (PA) were purchased from MedBio. Type I collagen was purchased from SolarBio. Sixty noncaries and intact human third molars were collected with the approval of the Institutional Review Board of the last author's institute (#IRB‐S2021‐655‐01). All extracted teeth were used within 1 month of extraction.

Twenty‐six dentin slices (10 mm diameter, 1 mm thickness), 20 dentin beams (1 × 1 × 6 mm), and 310 mg of dentin powder were prepared as previously described (Xu et al., 2019). All specimens were demineralized in 10 wt% PA for 24 h and then rinsed with deionized water for 20 min to prepare dental collagen. Aloin solution (0.1 mg/mL) was prepared by mechanical stirring and ultrasonic agitation for 10 min each. The tested concentration was based on the natural proportion of aloin in aloe. To evaluate the effects of aloin, half of the randomly selected specimens of demineralized dental collagen were used as controls (DDC group). The other specimens were treated with the aloin solution for 2 min and then rinsed with deionized water for 1 min. Aloin‐treated dental collagen was used in the experimental group (A‐DDC group).

### 2.2 The influence of aloin on dental collagen structure

#### 2.2.1 Attenuated total reflectance Fourier transform infrared spectroscopy (ATR‐FTIR) and peak fitting

ATR‐FTIR (Shimadzu) was used to explore the in‐situ interactions between aloin and dental collagen. Demineralized dental collagen slices were used as specimens and placed on the ATR‐FTIR diamond crystal top plate with a gauge force of 100 N. The characteristic peaks of amides I, II and III in the DDC and A‐DDC groups were recorded. The wavelength range was set at 400–4000 cm^−1^ with a resolution of 2 cm^−1^. Background spectra were collected with no sample on the ATR crystal top plate, and this spectrum was subtracted from all the absorbance spectra. Gaussian peak fitting was used to quantify the content of each secondary structure of dental collagen (*n* = 3) (Bridelli et al., 2017).

#### 2.2.2 X‐ray diffraction

The crystallinity index of dental collagen specimens was analyzed with X‐ray diffraction (XRD) (Bruker D8) using CuKα radiation (40 kV, 40 mA). The samples were scanned from 0° to 70°.

#### 2.2.3 Fluorescence spectroscopy

Fluorescence measurements were conducted under ambient‐temperature conditions using the FLS1000 (Edinburgh Instruments) with the excitation wavelength set at 280 nm and a scanning range of 200–700 nm. Type I collagen was incubated with aloin solution, and the fluorescence spectra were measured in triplicate with subsequent averaging of the values. Additionally, reference spectra were obtained for the solubilized collagen and aloin solutions.

### 2.3 Mechanical stability

The ultimate tensile strength (UTS) before and after thermocycling was measured to evaluate the effect of aloin on the mechanical stability of dental collagen. Twenty dental beams were used in the study. Half of the randomly selected specimens were used to evaluate the immediate UTS. Thermocycling was used to evaluate the effect of aloin on the stability of the other half of the dental collagen according to the ISO TR 11450 standard (1994). All specimens were tested at a 1 mm/min crosshead speed using a microtensile testing machine (EZ‐TEST 500 N; Shimadzu). The maximum load (*N*), length (*L*), and width (*W*) of the cross‐sectional areas were measured when the specimens failed. The UTS (*E*) of each group was calculated using the formula *E* = *N*/(*L* × *W*) (*n* = 5).

### 2.4 Thermostability

Forty‐eight milligrams of demineralized dentin powder was randomly divided into two groups. For each group, 8.0 mg of powder was initially used and dried in a vacuum desiccator with silica gel for 24 h before thermogravimetric analysis (TGA; Mettler Toledo) from 25 to 300°C at a heating rate of 10°C/min under N_2_ atmosphere (flow rate = 10 mL/min). The 5% mass‐loss temperature (*T*_95%_) was recorded (*n* = 3).

### 2.5 Enzymatic stability

#### 2.5.1 Release of cross‐linked carboxy‐terminal telopeptide and C‐terminal cross‐linked telopeptide of type I collagen (ICTP, CTX)

For each group, 5 mg powder was immersed in 2 mL artificial saliva at 37°C to simulate the influence of the oral environment for 7 and 14 days. The culture solution was collected and the amounts of ICTP and CTX were measured separately using ICTP (Finetest) and CTX ELISA kits (IDS, England). Standard curves were plotted before measurement according to the manufacturer's instructions. OD 450 nm was recorded and the concentrations of ICTP and CTX were calculated based on a standard curve (*n* = 5).

#### 2.5.2 Release of hydroxyproline

Hydroxyproline content in the extract is a reliable indicator of collagen degradation. High‐performance liquid chromatography (Agilent Technologies) measured HYP release in each group (*n* = 3). Two groups of demineralized dentin powders (5 mg) were immersed in 2.5 mL collagenase solution (50 μg/mL; Invitrogen) for 24 and 48 h. The detailed procedure was the same as described (Yu et al., 2020).

#### 2.5.3 Weight loss

Twenty slices from the DDC and A‐DDC groups were dried, as mentioned in Section 2.4, and weighed (*m*_1_). Then, the specimens were immersed in collagenase solution (50 μg/mL) for 7 days and 14 days, respectively. The specimens at the corresponding time points for each group were washed, dried until the mass remained unchanged, and weighed (*m*_2_). The weight loss (*W*%) was described by the percentage weight loss of individual samples, calculated by the following formula: *W*% = (*m*_1_ − *m*_2_)/*m*_1_ × 100% (*n* = 5).

### 2.6 Statistical analysis

After normal distribution and variance evaluation by D'Agostino–Pearson and Fisher's tests, the mechanical stability and enzymatic stability data were subjected to a two‐way analysis of variance and Bonferroni's repeated measures with Graph Pad Prism (version 8.0). Student's *t*‐test was used to compare *T*_95%_ and secondary structure parameters in the curve fitting. The significance level was set at *p* < .05.

## 3 RESULTS

### 3.1 The influence of aloin on dental collagen structure

**FIGURE 1:**
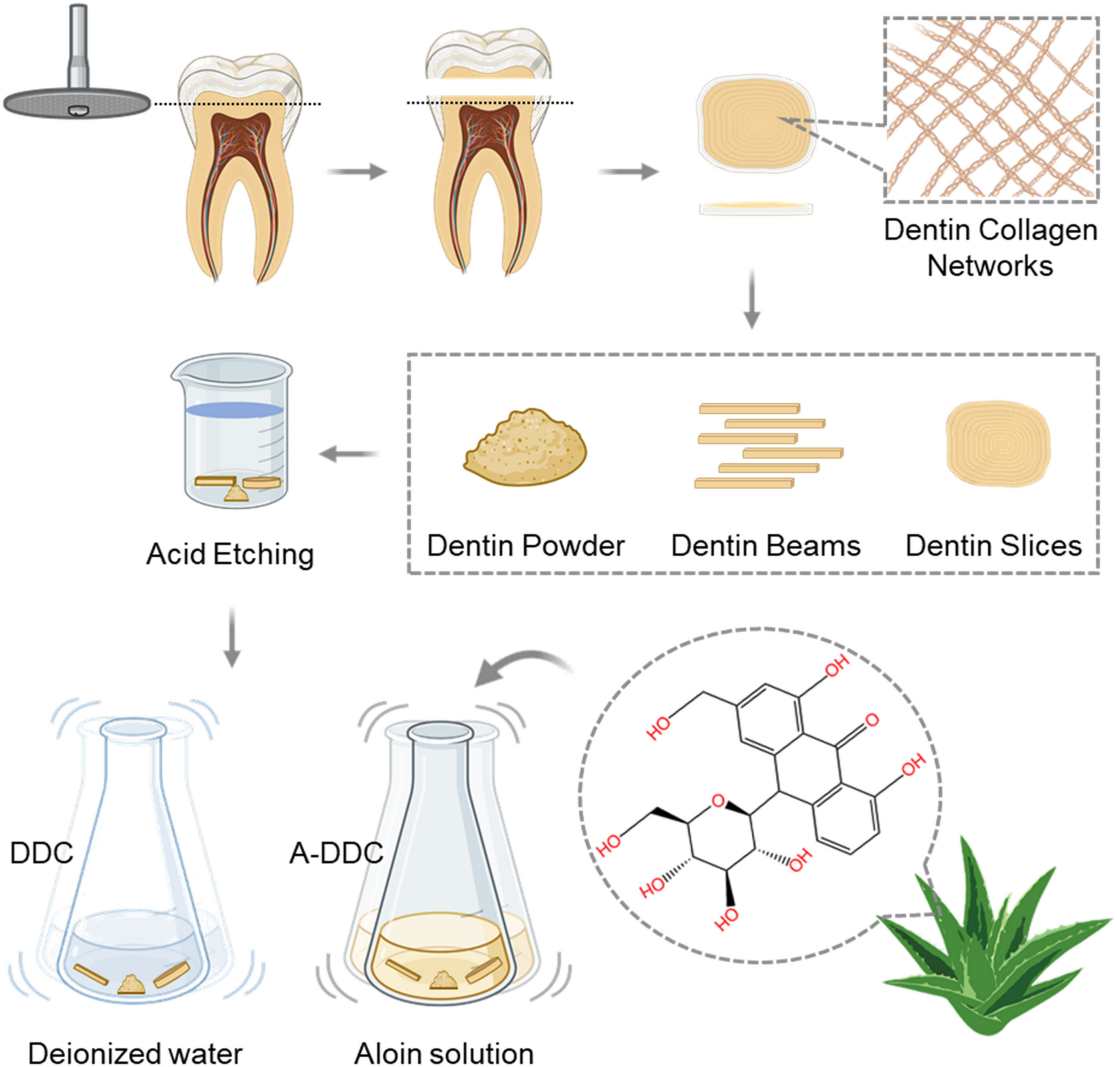
Schematic diagram of the preparation of demineralized dental collagen and A‐DDC.

**FIGURE 2:**
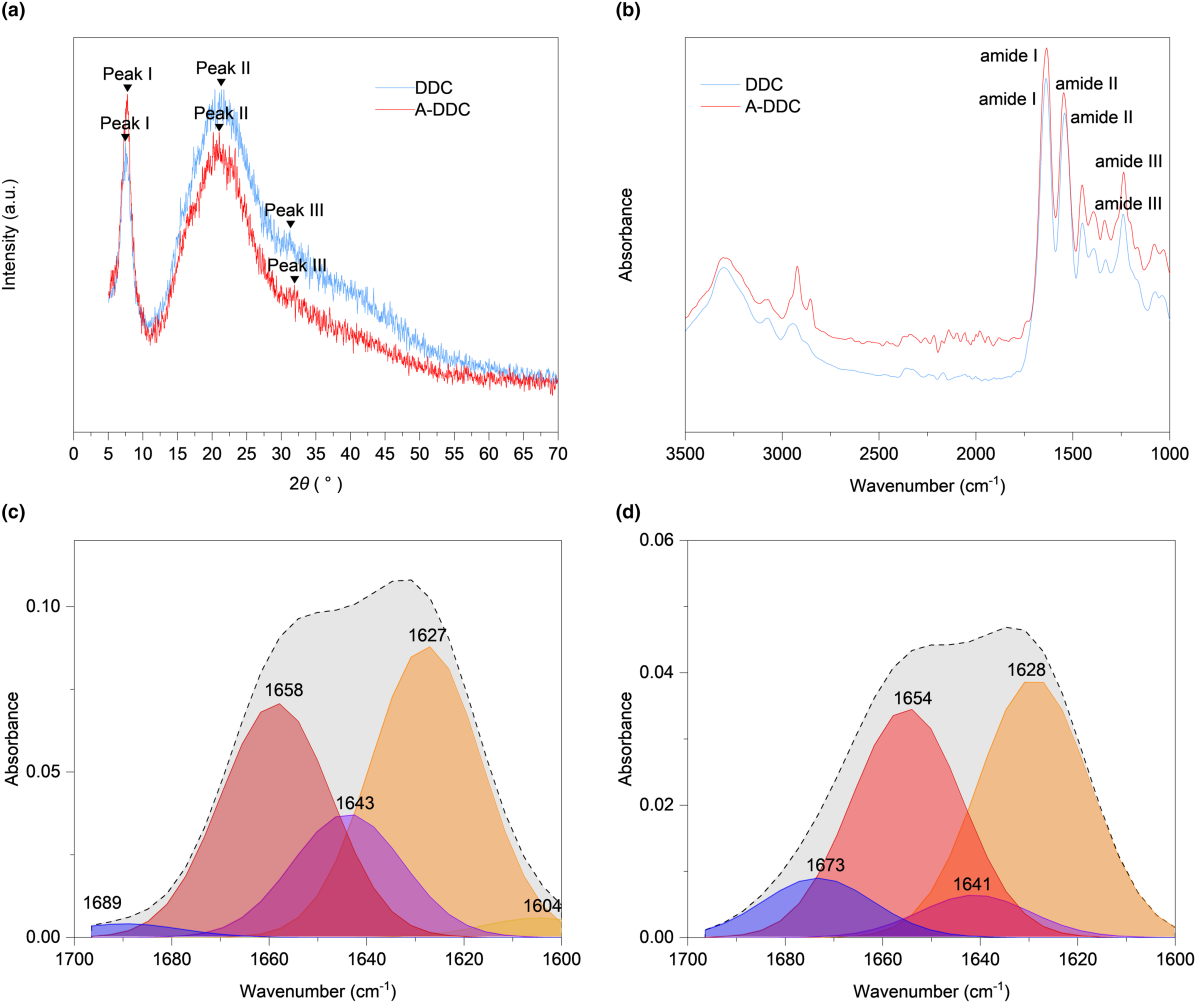
Interaction between aloin and demineralized dentinal collagen matrix. (a) X‐ray diffraction pattern and (b) infrared spectra of demineralized dental collagen (DDC) and A‐DDC. Gaussian deconvolution of the characteristic peak located in the 1600–1700 cm^−1^ of DDC (c) and A‐DDC group (d), respectively.

A schematic diagram of the dental collagen preparation and modification process is shown in Figure 1. Both DDC and A‐DDC exhibited the typical XRD spectra of dental collagen (Figure 2a). The DDC showed XRD peaks at 2*θ* = 7.51° (*d* = 11.76), 21.01° (*d* = 4.23), and 31.21° (*d* = 2.86). The A‐DDC exhibited three characteristic peaks at 2*θ* = 7.56° (*d* = 11.68), 20.80° (*d* = 4.27), and 31.61° (*d* = 2.83). An increased intensity of peak I and decreased intensities of peaks II and III were detected in A‐DDC compared to DDC.

The ATR‐FTIR spectra of both DDC and A‐DDC showed characteristic amide I, amide II, and amide III peaks of dentin collagen (Figure 2b). After modification with aloin, the amide I peak displayed a blue shift from 1639.38 to 1635.52 cm^−1^. Meanwhile, the spectral intensity of A‐DDC showed a slight decrease compared with that of DDC, further confirming the interaction between aloin and dental collagen.

**FIGURE 3:**
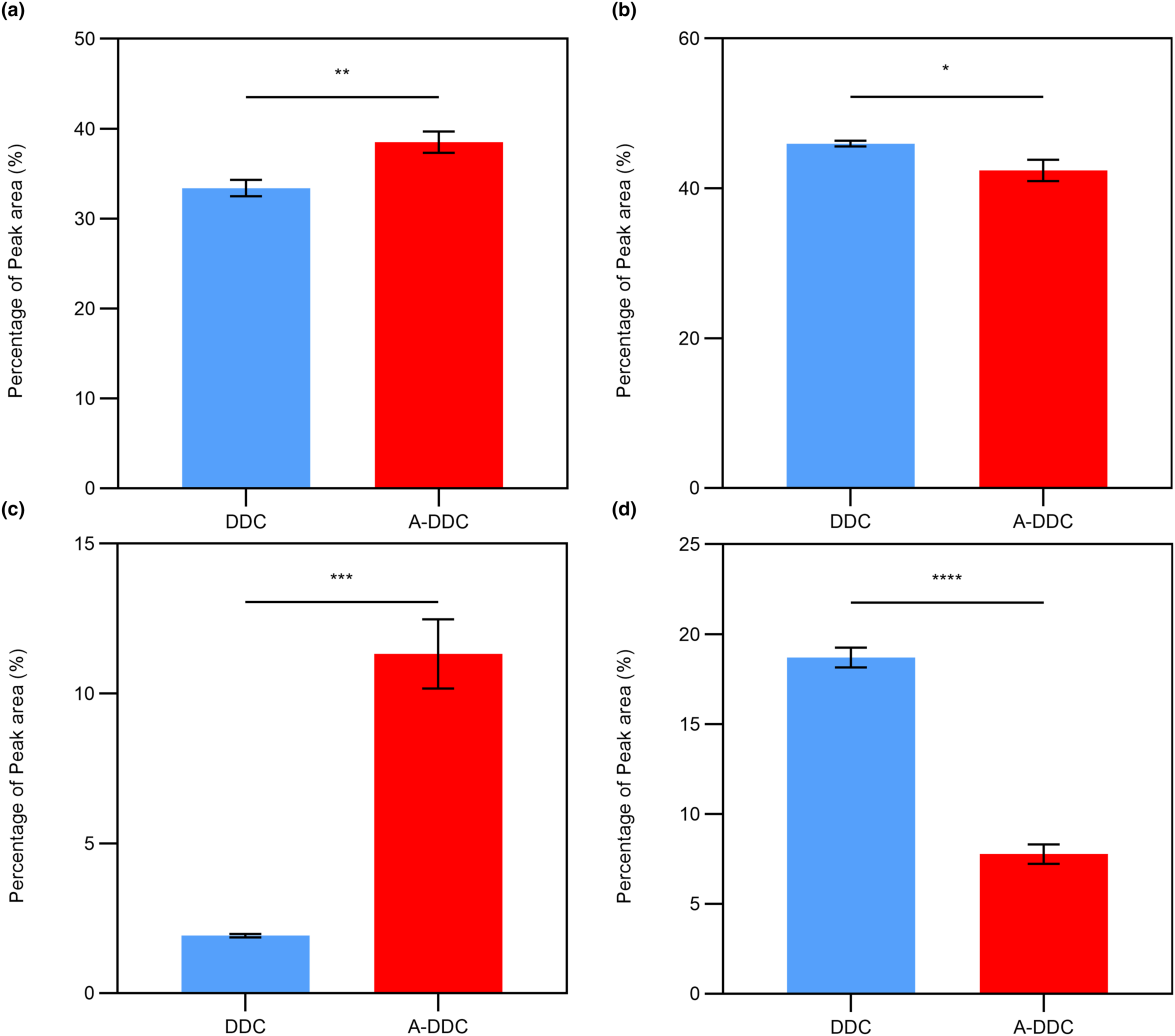
The percentage of secondary structure of demineralized dental collagen and A‐DDC. (a) The content of α‐helix, (b) the content of β‐sheet, (c) the content of β‐turn, and (d) the content of random coil. (Data are presented as the mean ± standard deviation (SD); *p < .05; **p < .01; ***p < .001; ****p < .0001.)

The curve fitting procedure allowed determining the secondary structure composition in both groups (Figure 2c,d): α‐helix (1650–1660 cm^−1^), β‐sheet (1600–1640 cm^−1^), β‐turn (1660–1700 cm^−1^), and random coil (1640–1650 cm^−1^). The intensity of the signals decreased after pretreatment with aloin compared with DDC. The subgroup distribution of the secondary structure was identical, but the proportions differed after modification. As shown in Figure 3a–d, the proportion of α‐helix increased from 33.40 ± 0.90% to 38.51 ± 1.17% (*p* < .01), and the proportion of random coil decreased from 18.70 ± 0.56% to 7.78 ± 0.54% (*p <* .0001).

**FIGURE 4:**
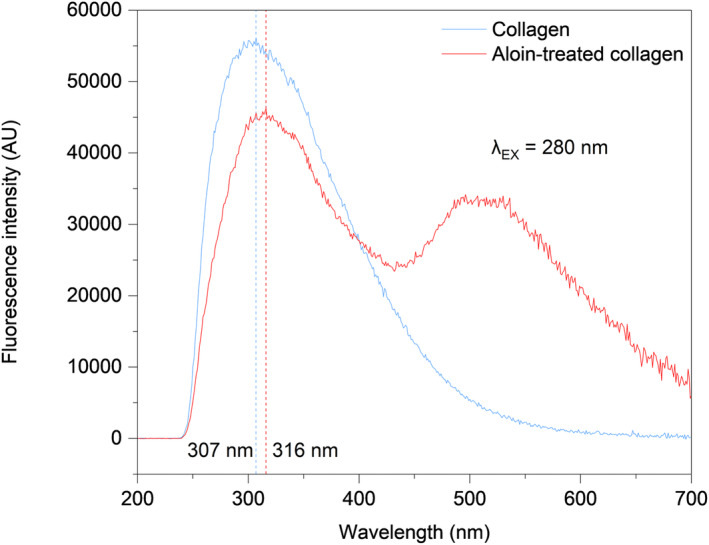
Fluorescence emission spectra of pure and aloin‐treated collagen, respectively.

The mean fluorescence spectra of the two groups are shown in Figure 4. Notably, when excited at 280 nm, type I collagen emits fluorescence at approximately 307 nm, characteristic of tyrosine (Tyr) fluorescence (Li, Cai, et al., 2020; Usha et al., 2009). Treatment of type I collagen with aloin resulted in a shift in the peak from 307 to 316 nm, and a notable reduction in fluorescence intensity. Furthermore, the peaks observed in the range of 496–536 nm can be attributed to the fluorescence exhibited by aloin.

### 3.2 Mechanical stability

**FIGURE 5:**
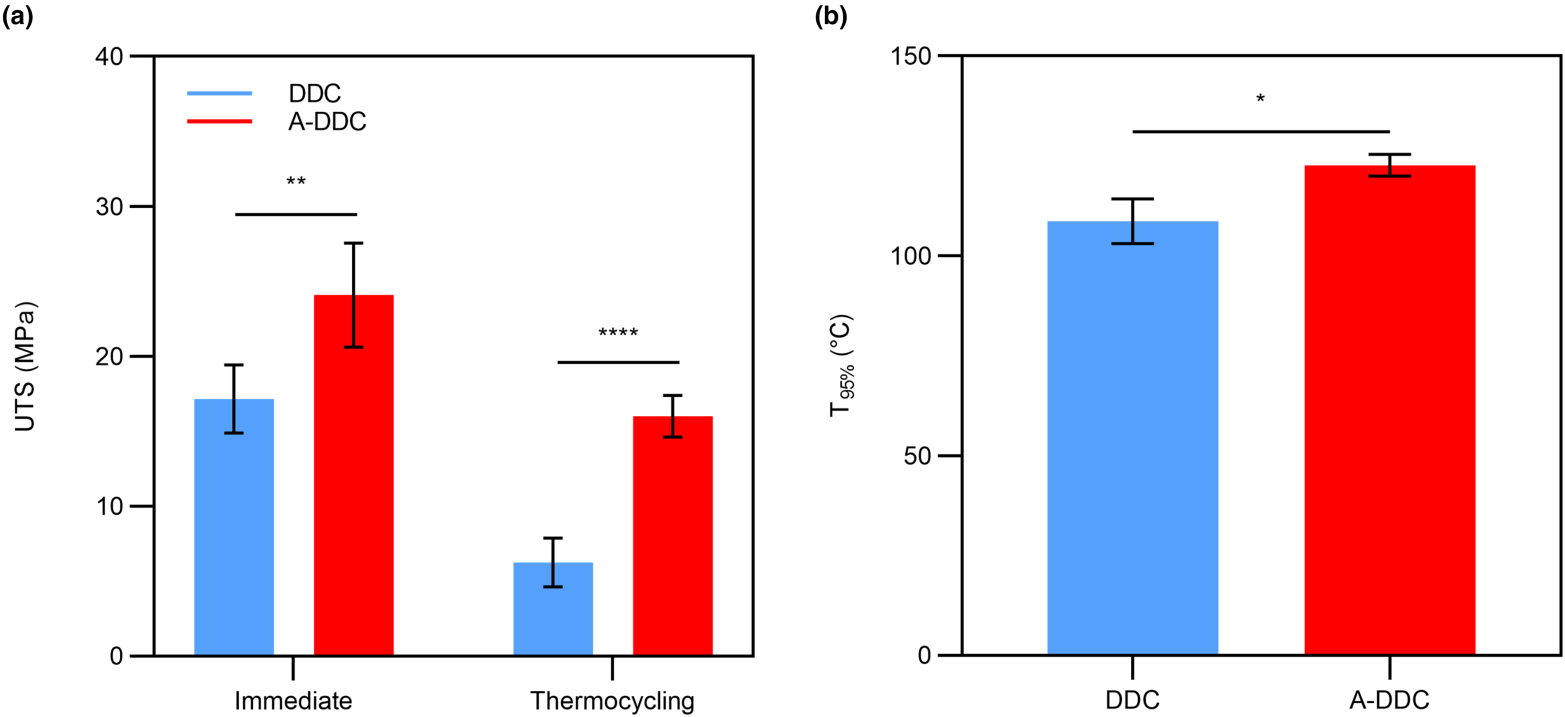
The mechanical strength (a) and thermostability (b) of each group. (Data are presented as the mean ± SD; *p < .05; **p < .01; ****p < .0001).

The UTS results are presented in Figure 5a. For immediate UTS, a significant difference was noted in A‐DDC (24.09 ± 3.47 MPa) compared with DDC (17.16 ± 2.26 MPa) (*p* < .01). There was a sharp decrease in UTS in both groups following the thermocycling protocol. While A‐DDC presented a 33.62% decrease in UTS, the value (15.99 ± 0.39 MPa) remained significantly higher than that of DDC (6.24 ± 1.23 MPa) (*p* < .0001).

### 3.3 Thermostability

The thermostability of the aloin‐treated collagen was evaluated using TGA. As shown in Figure 5b and Figure S2, *T*_95%_ of the treated collagen was higher than that of the untreated collagen (*p* <.05). For DDC, *T*_95%_ was 108.60 ± 5.60°C. After treatment with aloin solution for 2 min, *T*_95%_ increased significantly to 122.70 ± 2.79°C.

### 3.4 Enzymatic stability

#### 3.4.1 Release of ICTP and CTX

**FIGURE 6:**
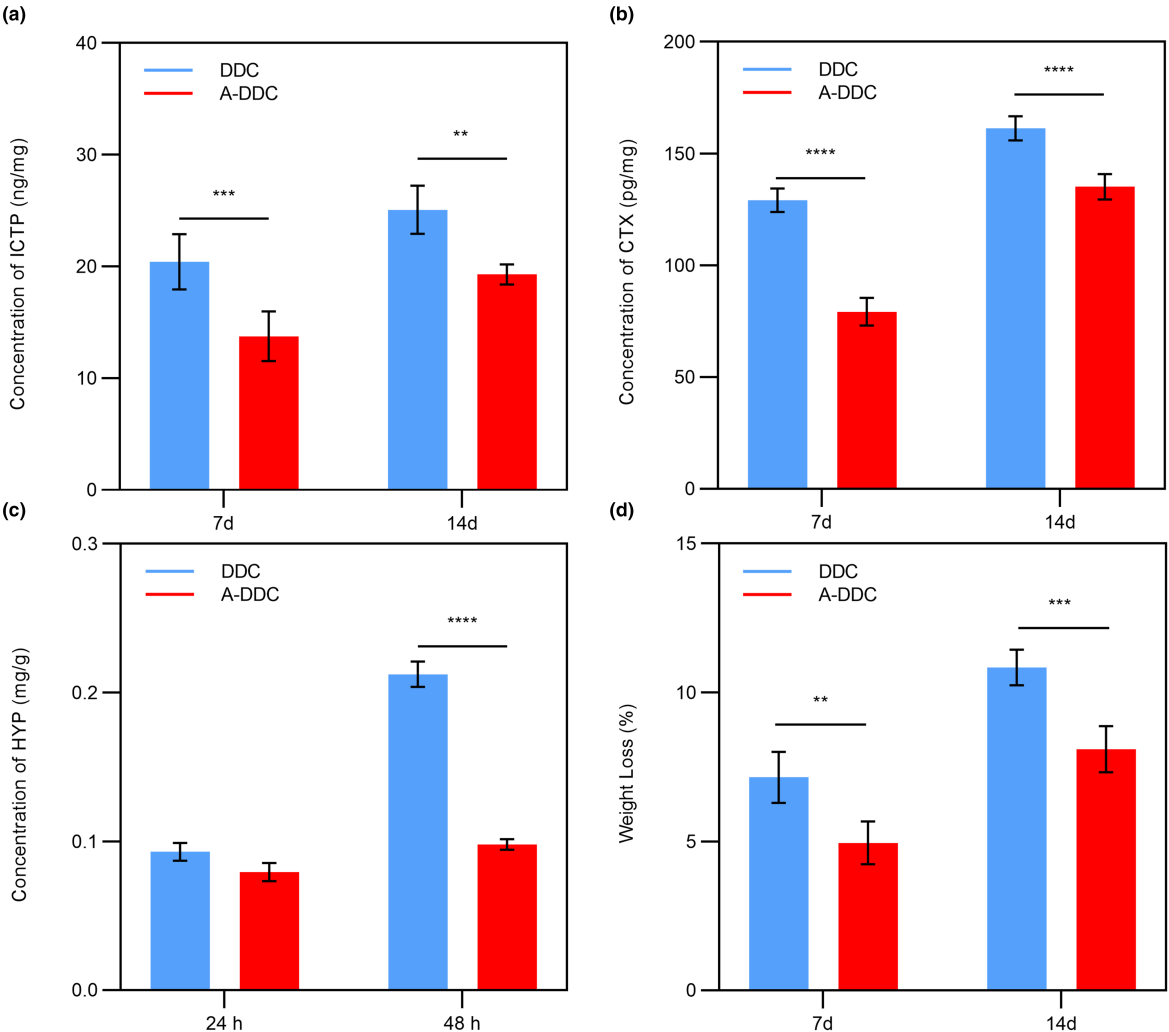
The enzymatic stability of demineralized dental collagen before and after treatment with aloin. (a) the concentration of ICTP, (b) the concentration of CTX, (c) the concentration of HYP, and (d) the weight loss for each group. (Data are presented as the mean ± SD; **p < .01; ***p < .001; ****p < .0001.)

Figure 6a summarizes the release of telopeptide ICTP by endogenous MMPs in dentin. The mean liberation of ICTP was 20.42 ± 2.48 ng/mg dry dentin for 7 days in DDC, and 13.76 ± 2.21 ng/mg dry dentin in A‐DDC (*p* < .001). The ICTP content in both groups was higher after 14 days than after 7 days (*p* < .05). A‐DDC still showed a significantly lower rate of ICTP telopeptide release for 14 days (19.30 ± 0.89 ng/mg dry dentin) than DDC (25.08 ± 2.15 ng/mg dry dentin) (*p* < .01).

Figure 6b shows the release of telopeptide CTX produced by cathepsin K. Similar to ICTP, the A‐DDC showed CTX telopeptide release both for 7 days (79.24 ± 6.16 pg/mg dry dentin) and 14 days (135.20 ± 5.73 pg/mg dry dentin); however, it was significantly lower than that in DDC (129.10 ± 5.30 and 161.40 ± 5.41 pg/mg dry dentin for 7 and 14 days, respectively) (*p* < .0001).

#### 3.4.2 Release of HYP

Resistance to collagenase digestion was determined by measuring the free HYP levels. As shown in Figure 6c, no significant difference was observed for 24 h between A‐DDC (0.08 ± 0.01 mg/g dentin) and DDC (0.09 ± 0.01 mg/g dentin) (*p* = 0.17). After 48 h, there was significantly lower release of HYP in A‐DDC (0.10 ± 0.00 mg/g dentin) compared to DDC (0.21 ± 0.01 mg/g dentin) (*p* < .0001).

#### 3.4.3 Weight loss

Figure 6d shows the weight loss of the demineralized dentin after 7 and 14 days. The A‐DDC showed a W% of 4.95 ± 0.72% for 7 days, which was lower than that in DDC (7.15 ± 0.86%, *p* < .01). The W% of both groups increased with time, and it was significantly lower in A‐DDC (8.10 ± 0.77%) compared to DDC (10.84 ± 0.60%) for 14 days (*p* < .001).

## 4 DISCUSSION

In the present study, we developed a new noninvasive dietary strategy for enhancing dental collagen stability using aloin. These results indicated that this strategy can significantly enhance the mechanical strength, thermostability, and resistance of dental collagen to enzymatic hydrolysis. This improvement was achieved through interactions between aloin and dentin collagen, particularly via hydrogen bonding. Hence, the null hypothesis is rejected.

Plant‐derived polyphenols, such as procyanidins, have been suggested as modifiers that can enhance the stability of collagen (Li, Yao, et al., 2021; Li, Zhang, et al., 2021; Mendes et al., 2020; Reis et al., 2021). Previous studies have suggested that plant‐derived polyphenols interact with collagen through covalent bonds, hydrogen bonds, and van der Waals forces (Li et al., 2022; Wang et al., 2023). The decrease in the fluorescence intensity of Tyr residues indicates fluorescence quenching during collagen–aloin interactions. Conformational changes caused by denaturation or ligand binding can mask the fluorescence intensity (Liu, Hao, et al., 2021; Liu, Lv, et al., 2021). However, the disappearance of the positive band was not observed in aloin‐treated collagen according to the CD spectra (see Figure S1), illustrating the presence of undamaged triple‐helix collagen molecules (Kalirajan et al., 2022). Moreover, adding aloin led to a red shift in the fluorescence peak frequencies. One possibility is that the interaction between the Tyr side chains and the catechol component of aloin results in the entrapment of Tyr residues in a local conformation. This entrapment allows Tyr residues to participate in events, such as excited‐state proton transfer, ultimately resulting in fluorescence emission with a red‐shifted wavelength (Vidal et al., 2016). Another possible explanation is the exposure of Tyr residues, which are typically protected within the hydrophobic core, to more polar and hydrophilic environments (Wang et al., 2021). Consequently, the microenvironment surrounding these Tyr residues became less hydrophobic. This suggests that the interaction between collagen and aloin may have occurred in the hydrophobic region of the Tyr residues.

Previous studies have indicated that the blue shift in the amide I band in the FTIR spectra can be explained by the formation of hydrogen bonds between the additive and dentin collagen (Chen et al., 2021). Based on the presence of catechol groups in the structure of aloin, the blue shift of the amide I peak from 1639.38 to 1635.52 cm^−1^ in ATR‐FTIR suggests that aloin may interact with dental collagen through hydrogen bonds. This finding is consistent with previous studies on other plant polyphenols (Omar et al., 2022). The decrease in the intensity of the amide II absorption band in A‐DDC suggested the transformation of more free –NH_2_ into N–H groups owing to collagen cross‐linking (Zhang et al., 2022). Moreover, these findings provide a potential explanation for the observed results in the amide I band, further indicating the formation of hydrogen bonds between the free amino group and the catechol structure in aloin. The amide III band was examined to assess the integrity of the collagen triple helix, and any changes in this band indicated conformational changes in the protein (Liu, Luo, et al., 2022; Liu, Zong, et al., 2022). In the A‐DDC group, a decrease in absorbance and a blue shift (from 1242.07 to 1238.22 cm^−1^) in the amide III region, suggesting modifications to the β‐sheet structure of the peptide (Jie et al., 2019). This hypothesis was also confirmed by curve fitting of amide I using FTIR.

In proteins, the amide I vibration is primarily influenced by the secondary structure of the backbone rather than by variations in the side‐chain structures (Huang et al., 2022). Consequently, curve fitting of the amide I peak was employed to study the secondary structure. As the most stable secondary structure in collagen type I, an increase in the content of α‐helix demonstrates the enhancement of dental collagen stability in the A‐DDC group (Qi et al., 2022). Meanwhile, the proportion of relatively unstable structures like β‐sheet, especially random coil, significantly decreases after treatment compared to the DDC group, confirming that the interactions between aloin and dental collagen affect the secondary structure (Yu, Khare, et al., 2022; Yu, Bian, et al., 2022; Yu, Zhao, et al., 2022). The proportions of secondary structures obtained from peak fitting and circular dichroism spectroscopy exhibited similar trends (see Table S1) despite the use of different samples, indicating the effectiveness of peak fitting for protein secondary structure analysis. As illustrated in Figure 2a, peak I intensities were higher in the XRD pattern of the A‐DDC group, suggesting that aloin may induce a shorter lateral spacing between collagen molecules (Green et al., 2017). This conjecture is further supported by the observed increase in 2*θ* angles of the diffraction peak I and III in the A‐DDC group, indicating that the addition of aloin reduces the spacing between collagen molecules (Ding et al., 2019; Nwambaekwe et al., 2021). Peak II, which can be seen at roughly 2*θ* ~20°, is due to the diffusive scattering caused by the presence of random‐coil structures embedded within the collagen fibers (Giraud‐Guille et al., 2000). In the A‐DDC group, peak II intensity decreased from 117 to 102, indicating a reduction in the number of random coils. This observation aligns with the results obtained from the peak fitting. The third peak at 2*θ* ~31° represents the height of the collagen triple helix (Labaki et al., 1991). In the A‐DDC group, the intensity of peak III decreased. Considering the results of peak fitting, it can be inferred that aloin interacts with collagen by converting more random coils into short but stable structures. This transformation led to a reduction in the average collagen height. Furthermore, the intensity of peaks II and III decreased with the addition of aloin, also suggesting the decrease in the structural order due to the presence of cross‐links (Andonegi et al., 2020). Combining the results of ATR‐FTIR, fluorescence spectroscopy, CD spectroscopy, and XRD, our findings suggested the presence of interactions between collagen and aloin. These interactions, particularly hydrogen bonding, lead to alterations in the secondary structure of dentin collagen.

The intraoral temperature, bite force, and enzymes play significant roles in determining the fate of dentin (Gale & Darvell, 1999). Hence, we chose UTS, TGA, and enzymatic degradation tests to assess the effect of aloin on the stability of dental collagen. The increased UTS and *T*_95%_ in the A‐DDC group showed the improved stability of dentin treated with aloin (Li, Yao, et al., 2021; Li, Zhang, et al., 2021). This improvement may result from the interactions between aloin and collagen, which enhance cross‐linking between collagen molecules and their structural stability. In addition, the enhanced thermostability could be attributed to aloin through several mechanisms, including increasing the proportion of stable secondary structures, augmenting the molecular weight of the reaction system, and facilitating the cross‐linking of thermally stable domains in dentin collagen (Miles & Bailey, 2001; Zhang et al., 2019). However, this last speculation was not investigated further in this study. The current literature lacks studies on the application of aloin to dental collagen, with aloe vera (AV) being the most commonly examined extract. In a previous study, the immediate UTS of dentin increased by 109.27% in the AV group (Sinha et al., 2016). Although this trend was consistent with our work, it was not as effective as aloin (140.38%) in improving the mechanical strength of dentin. This discrepancy could be attributed to variations in the number of catechol groups in the two substances and the treatment duration. Another study reported that AV also increased the tensile strength of alginate films by approximately 133.33% in tissue engineering applications, indicating the potential application value of aloin in future tissue engineering scaffolds (Koga et al., 2018). Contrary to our present study, Andonegi et al. (2020) reported a slight decrease in tensile strength when AV was added to collagen membranes (Andonegi et al., 2020). This inconsistency could be attributed to the rigid structure of AV, which restricts the mobility of polypeptide chains within the collagen membrane, as well as inherent differences between collagen membranes and dentin. By investigating the effect of aloin on dental collagen, our study provides insights into its potential use in dentistry and enhances our understanding of the diverse biological activities of various aloe components.

These findings prove that aloin can resist collagenase, as measured by the release of ICTP, CTX, and HYP and confirmed by weight loss after enzymatic hydrolysis. HYP is released due to the action of bacterial collagenase and other dentine‐bound proteases, indicating the tissue collagen concentration (Daood et al., 2019). The release of ICTP and CTX fragments represents specific degradation products resulting from MMPs and cathepsin K‐mediated activities (Bafail et al., 2019). Weight loss is a macroscopic manifestation of enzyme degradation (Hass et al., 2021). Effective inhibition of collagenase by aloin was observed in our study for several reasons. First, the stabilized collagen matrix acts as a mechanical barrier, preventing the unwinding of the triple helix, which is necessary for the cleavage of the catalytic site of collagenase. Second, aloin promotes conformational changes in collagen and alters the configuration of the active site of collagenase, rendering the protease unable to identify the complementary peptide sequence for collagen. Furthermore, similar to other plant‐derived polyphenols, aloin may exert additional effects directly on collagenase via chelation and hydrophobic interactions, reducing the molecular mobility of collagenase and altering the 3D structure of its catalytic or allosteric domains. The thermostability and enzymatic stability observed in our study are consistent with previous research on plant‐derived polyphenol (Omar et al., 2022; Reis et al., 2021; Zhao et al., 2022). A notable advantage of aloin over other plant‐derived polyphenols is its edibility, which enables the development of food‐based regulation strategies for dentin.

Previous research has demonstrated that phenols can interact with the intraoral microbiota, leading to the degradation of their activity and enhancement of the antibacterial capacity of dentin collagen (Iqbal et al., 2020). However, it is still under investigation whether aloin exhibits a similar effect. Future studies should be designed to quantify the in situ reaction kinetics to further explore the daily intake of aloin. Additionally, incorporating aloin into oral hygiene products such as mouthwashes and toothpastes may offer significant benefits, especially for individuals more prone to infectious oral diseases. Furthermore, adding aloin to dental adhesive resins can improve the stability of the dentin–resin interface, also known as the bonding interface. This enhanced stability ultimately leads to a more effective long‐term therapeutic effect of the resin composite. All these potential industrial applications will be explored in future research.

## 5 CONCLUSION

This study revealed that the aloin extracted from aloe significantly enhanced the mechanical strength, thermostability, and resistance to enzymatic degradation of dentin collagen. Furthermore, this study revealed that aloin interacted with collagen through noncovalent interactions, particularly hydrogen bonding, facilitating the cross‐linking of collagen molecules. Our findings provide a theoretical basis for developing foodborne prophylactic strategies. By enhancing dentin stability, this routine, nondestructive, and cost‐effective strategy shows promise for reducing the risk of infectious oral diseases.

## AUTHOR CONTRIBUTIONS

**Chongzhi Jia:** Formal analysis (equal); investigation (equal); methodology (equal); validation (equal); visualization (equal); writing – original draft (equal). **Hua Li:** Formal analysis (equal); investigation (equal); methodology (equal); validation (equal); writing – original draft (equal). **Zhongliang Yang:** Formal analysis (equal); investigation (equal); methodology (equal); validation (equal); writing – original draft (equal). **Rongchen Xu:** Conceptualization (lead); data curation (lead); funding acquisition (lead); resources (lead); writing – review and editing (lead). **Lijun Wang:** Project administration (supporting); supervision (lead); writing – review and editing (lead). **Hongbo Li:** Funding acquisition (lead); project administration (lead); supervision (lead); writing – review and editing (lead).

## FUNDING INFORMATION

National Natural Science Foundation of China, Grant/Award Number: 82001110, 82071154, and 82271012.

## CONFLICT OF INTEREST STATEMENT

The authors declare no conflicts of interest.

## ETHICS STATEMENT

This study was performed in accordance with the Declaration of Helsinki and the study protocol was reviewed and approved by the Ethics Committee of the Chinese PLA General Hospital (#IRB‐S2021‐655‐01).

## CONSENT FOR PUBLICATION

All the authors of this manuscript gave their consent to publish.

## Supporting information


Appendix S1.

## Data Availability

All data generated or analyzed from this study are included in this published article.
