# Supplementary material for: From medical strategy to foodborne prophylactic strategy: Stabilizing dental collagen with aloin
Source: Food Sci Nutr. 2023 Nov 7;12(2):830–42. doi: 10.1002/fsn3.3795 (PMC10867467; doi:10.1002/fsn3.3795)
Supplement: Supplementary file 1 — Appendix S1. [file FSN3-12-830-s001.docx]

**MATERIAL S & METHODS** **for** **Circular dichroism (CD) spectra**

Circular dichroism (Jasco, J-815) was used to study the aloin-induced changes in the secondary structure of type I collagen. The CD spectra of the samples were recorded in a cuvette with a pathlength of 1 mm in the UV region (190−230 nm) at 25°C under a nitrogen atmosphere. In addition, native and aloin-treated type I collagen solutions were diluted to a specific concentration (0.1 mg/mL) and subjected to CD spectroscopic analysis. The sample scanning speed was 100 nm/min, and each sample was scanned 3 times on average.

**RESULTS for CD spectra**

The CD spectra revealed a positive peak at ~220 nm and a negative peak at ~198 nm in both samples. Additionally, the negative peak of the A-DDC solutions demonstrated a slight blue shift from 196.60 nm to 196.40 nm, and no redshifts were detected in the negative peak of the aloin-treated solution. The presence of aloin resulted in an increase in molar ellipticity at negative peak in the aloin-treated collagen, along with a corresponding decrease at positive peak. The secondary structure of collagen underwent slight changes when combined with aloin. The quantitative analysis of collagen's secondary structure was presented in Table 1. Pure collagen consisted of 4.10% α-helix, 2.30% β-sheet, 3.90% β-turn, and 89.70% random coil. Upon adding aloin at a concentration of 0.10 mg/mL, the α-helix content increased from 4.10% to 8.50%, while the β-turn content decreased from 2.30% to 1.20%, and the random coil content decreased from 89.70% to 82.70%.





**SUPPLEMENTARY FIGURE 1 Circular dichroism spectra of pure and aloin-treated collagen, respectively.**

| Samples | Secondary structure data | | | |
| --- | --- | --- | --- | --- |
|  | α-helix (%) | β-sheet (%) | β-turn (%) | random coil (%) |
| Collagen | 4.10% | 2.30% | 3.90% | 89.70% |
| Aloin-treated Collagen | 8.50% | 1.20% | 7.60% | 82.70% |

**SUPPLEMENTARY TABLE 1** The Secondary structure proportion for native collagen and aloin-treated collagen





**SUPPLEMENTARY FIGURE 2** Thermostability of dentin collagen treated with/without aloin.
